# Anti-inflammatory properties of a wound dressing combination of zinc oxide and turmeric extract

**DOI:** 10.14202/vetworld.2018.25-29

**Published:** 2018-01-17

**Authors:** Asti Meizarini, Aryati, Wibi Riawan, Astari Puteri

**Affiliations:** 1 Department of Dental Material, Faculty of Dental Medicine, Universitas Airlangga, Surabaya, Indonesia; 2 Department of Clinical Pathology, Faculty of Medicine, Universitas Airlangga, Surabaya, Indonesia; 3 Department of Biochemistry and Molecular Biology, Faculty of Medicine, Universitas Brawijaya, Malang, Indonesia; 4 Dentist, Surabaya, Indonesia

**Keywords:** cyclooxygenase-2, MAC387, turmeric, wound dressing, zinc oxide

## Abstract

**Aim:**

The aim of this study was to determine the effect of a wound dressing consisting of a zinc oxide with turmeric extract combination as an anti-inflammatory on the healing process through the expression of MAC387 and cyclooxygenase-2 (COX-2).

**Materials and Methods:**

Forty Wistar rats were divided into four control and four treatment groups (n=5). On day 1, a 6 mm×6 mm square of skin in the area of the vertebralis thoracis was excised from all subjects. The wound was then dressed with a combination of zinc oxide and turmeric extract for the treatment groups, while the control groups were left undressed. Both the control and treatment groups were then sequentially sacrificed on days 3, 5, 7, and 14 to obtain subepithelial excision samples. These samples subsequently underwent immunohistochemistry examination through the expression of MAC387 and COX-2 to ascertain the anti-inflammatory reaction to the wound healing process.

**Results:**

The highest expression of MAC387 was found in the treatment group to which a dressing of zinc oxide with turmeric extract had been applied on the day 5 before slowly reducing on days 7 and 14. MAC387 peaked in the undressed control group on day 14. The COX-2 expression results in control groups showed their higher expression on day 3, increased up to day 5, began to decline on day 7 before, and finally, decreasing on day 14. This result was different to those treatment groups which presented a high COX-2 expression on day 3, before gradually decreasing between days 5 and 7 and reaching its lowest point on day 14.

**Conclusion:**

A wound dressing consisting of a combination of zinc oxide and turmeric extract has been proven effective as an anti-inflammatory in the healing process.

## Introduction

The periodontal wound dressing is widely used in treating post-dental surgery wounds as it offers protection against mechanical trauma and maintains its stability during the healing process. Wound dressing promotes effective adaptation of gingival tissue and underlying bone, prevents bleeding or infection, decreases dental hypersensitivity in the 1^st^ h after surgery, protects blood clots from pressure during speech or mastication, and prevents gingival discharge from the tooth root surface. In general, the use of dressings for the closure of the periodontal surgical wound area lasts for 3–14 days [1,2].

A combination of eugenol or rosin and zinc oxide was commonly used as wound dressing material, but both eugenol and rosin were found to cause allergic reactions in some people [3,4]. A previous study conducted by Meizarini *et al*. [5], sought to combine zinc oxide with turmeric extract as an alternative material for wound dressings, showed this combination to be capable of accelerating the inflammatory stages of the wound healing process by increasing the expression of macrophages and lowering that of toll-like receptor 2, nuclear factor kappa B, and tumor necrosis factor-alpha.

Macrophages are essential to the wound healing process through their ability to release factors that attract inflammatory cells to the wound sites, stimulate granulation tissue formation, and enhance cell migration. Given the preference for obtaining more specific results when identifying macrophages by means of immunohistochemistry examination, MAC387 was used to identify macrophages in the network as it was more specific than the CD68 marker [6].

Cyclooxygenase-2 (COX-2) was originally discovered to be an increased transcription during the period of inflammation and cellular changes. Animal models with acute and chronic arthritic inflammation presented such enhanced COX-2 expression leading to an increase in prostaglandin production in inflamed joint tissues [7]. One mechanism of curcumin as an anti-inflammatory is the inhibiting of the COX-2 enzyme which decreases prostaglandin production (causing inflammation), thus accelerating wound healing [8].

The research reported here aims to determine the effect of a wound dressing combination of zinc oxide and turmeric extract as an anti-inflammatory in the healing process through the expression of MAC387 and COX-2. The result will be compared to that of the control group which experienced a similar excision but without a dressing of zinc oxide with turmeric extract.

## Materials and Methods

### Ethical approval

This laboratory-based research was conducted in accordance with the ethics governing the experimental use of animals approved by the Health Research Ethical Clearance Commission of the Faculty of Dental Medicine, Universitas Airlangga, No. 139/HRECC.FODM/VIII/2017.

### Preparation of turmeric rhizome liquid extracts

Turmeric rhizome material was supplied by and extracted at the Balai Materia Medica Batu, Department of Health of East Java Province. The fresh turmeric rhizome harvested from 10-month-old turmeric plants was washed with running water, drained, and cut into 4 mm thick slices. Completely dehydrated by 3 days of exposure to direct sunlight, it was ground to produce turmeric rhizome powder which was then macerated with 96% ethanol. 500 g of the powder was hydrated with 500 ml solvent (96% ethanol) in a jar, flattened and mixed with solvent until dissolved with 3 L ethanol. Tightly sealed, the jar was agitated by means of a 50 rpm digital shaker for 24 h. The liquid extract was passed through a cloth filter and accommodated in an Erlenmeyer tube. After remaceration with 3 L ethanol for 24 h, the results of the first and second extract were mixed together and subjected to a 6-h evaporation process using a 60°C rotary evaporator until all the ethanol solvents had been separated. Finally, the extract was placed in a bowl immersed in a 37°C water bath for 2 h.

### Animal

Forty male, Wistar strain *Rattus norvegicus* within the weight range of 200-300 g were purchased from Wistar Farm (Malang, Indonesia) and randomly divided into control and treatment groups each containing 4 subgroups (n=5). One subgroup from the control and treatment groups was sequentially sacrificed on day 3 (C3, T3), day 5 (C5, T5), day 7 (C7, T7), and day 14 (C14, T14). The rats were acclimatized for 1 week in eight cages made from plastic tubs covered with woven wire mesh wood subjected to 12 h of light followed by 12 h of darkness. Both before and during the study, observation was conducted of the behavior of the subjects (eating and drinking habits, mental and clinical conditions), as well as environmental conditions (temperature, humidity, cage conditions, ventilation, and pollution). The rats were fed *ad libitum* with commercial rodent chow and water.

After the adaptation period, each Wistar was anesthetized intramuscularly by means of a combination of ketamine (KEPRO, ZA, Denmark) and xylazine (Interchemie werken, Venray, Holland) at a ratio of 1:1 (0.1 ml/rat body weight) in the right groin. The skin around the area to be excised (vertebralis thoracis) was sterilized using 70% alcohol, shaved, and drawn with 6 mm×6 mm square as an excision pattern guide. An excision of full thickness, 6 mm long and 6 mm wide, of sufficient depth that the fascia became visible (about 2 mm), was made using a surgical blade No. 15 (Swann-Morton, Sheffield, England,), razor, scissors, and chirurgical tweezers [5].

### Wound dressing application

99.8% zinc oxide, catalog number 1.08849.0500, was purchased from Merck-Germany (Merck KGaA, Darmstadt, Germany). One part of zinc oxide powder (0.3 g) was mixed with one part of turmeric rhizome liquid extract (0.3 g) on a mixing pad for 60 s using a stainless steel spatula. The resulting mixture was applied as a dressing to the surface of the excision wound after the latter had been cleaned by the application of physiological saline solution [5].

### Wound management

The excision wounds in the control group were left undressed but covered by hypoallergenic tape (Hypafix, Germany), while the excision wounds of the treatment groups were dressed using a combination of zinc oxide with turmeric rhizome liquid extract (0.3 g:0.3 g) and covered with hypoallergenic tape. At the end of each period (days 3, 5, 7, and 14), the rats were euthanized by means of an overdose of anesthesia. The granulation tissues formed on the injury site were subepithelially excised with a 5 mm extended margin of normal skin for immunohistochemistry assessment.

### Preparation of immunohistochemistry staining specimens

The wound tissues were dehydrated with ethanol, clearing with xylol, impregnated with paraffin liquid, and embedded in the paraffin blocks, after they had been fixed in 10% neutral buffered formalin for 48 h. Paraffin blocks of 4 µm thickness were sectioned with a microtome, placed on a poly-L-lysine slide surface, and heated at 30-35°C on a hot plate for 24 h [9,10]. Before being immunohistochemically stained, each slide was washed by means of xylol, water, ethanol, and phosphate-buffered saline (PBS) pH 7.4 to achieve deparaffinization. Immunohistochemical examination was performed according to the avidin-biotin complex. The tissue was incubated with trypsin 0.125% at 37°C for 5-10 min to open the masking antigen, then incubated with hydrogen peroxide (H_2_O_2_) diluted in methanol for 30 min to remove endogenous staining, and washed with distilled water for 10 min. Non-specific binding of antibodies was blocked by means of incubation with normal goat serum (D-BioSys, The Hague, Netherlands) and PBS at a 1:4 dilution ratio. The sections were incubated with specific monoclonal antibody Macrophage Marker Antibody (MAC387) sc-66204 (Santa Cruz Biotechnology, Dallas, USA) and COX-2 (29) sc-19999 (Santa Cruz Biotechnology, Dallas, USA), diluted in fetal bovine serum with 1:100 ratio, for 1 h at room temperature. The sections were subsequently washed in PBS pH 7.2 and incubated with biotinylated universal secondary antibody (D-BioSys, The Hague, Netherlands), before being washed again in PBS pH 7.2. The sections were incubated with Strep-Avidin HRP conjugated (D-BioSys, The Hague, Netherlands) and washed in PBS. Peroxidase was detected with a diaminobenzidine (DAB) substrate kit (Histofine-Nichirei Bioscience, Tokyo, Japan), washed in tap water for 10 min, and then dehydrated. The nuclei were stained with hematoxylin and the sections mounted with Entellan (Merck KGaA, Darmstadt, Germany). PBS was used for all dilutions and thorough washes unless otherwise specified [5].

### Immunohistochemistry expression

The microscope slides were initially viewed under a 100× light magnification microscope (Nikon Eclipse E 100, Tokyo, Japan) to observe the entire field of view, before being enhanced at 400× and 1000× magnification. The area to be observed was first determined. The expression of positive MAC387 and COX-2 will appear brown under examination by a 1000× magnification microscope. The calculation was performed at 1000× enlargement in 20 specific fields of view, performed by two researchers with 95% clinical agreement. Pictures were taken with a Sony ILCE α6000 camera (Sony, Tokyo, Japan).

### Statistical analysis

The data were analyzed by means of SPSS version 21 (IBM, New York, USA) and results were expressed as mean ± standard deviations. One-way analysis of variance (ANOVA) followed by a Tukey test or Kruskall–Wallis followed by Mann–Whitney was applied to assess the statistical significance of the differences between the study groups at p<0.05.

## Results

**Figure-1:**
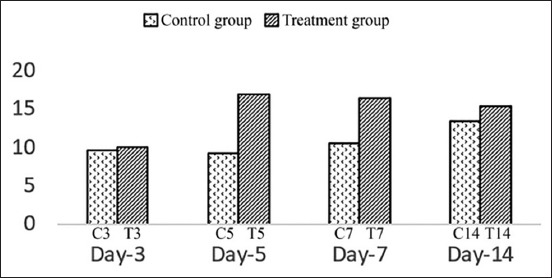
The MAC387 expression in control groups and treatment groups on day 3, 5, 7, and 14.

**Figure-2:**
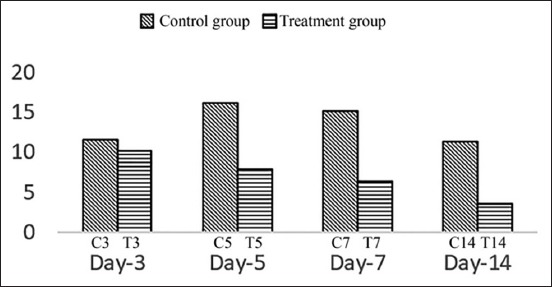
The cyclooxygenase-2 expression in control groups and treatment groups on day 3, 5, 7, and 14.

Statistical analysis of MAC387 was performed using ANOVA followed by a Tukey test because data distribution was found to be normal and homogenic, while for COX-2, Kruskal–Wallis followed by Mann–Whitney was employed because the data distribution was normal, although not homogenic. The result variables of this research were MAC387 and COX-2 expressions (Table-1). The expression of MAC387 (Figure-1) was highest in the treatment group on day 5 (T5) and slowly reduced on days 7 (T7) and 14 (T14). In contrast, macrophages in the control groups peaked on day 14 (C14). The inflammatory process, characterized by high COX-2 expression (Figure-2), was identified in control group on day 3 after excision (C3) and continuing to increase until day 5 (C5), before declining on day 7 (C7) and decreasing further on day 14 (C14). In contrast, in the treatment group, the expression of COX-2 gradually decreased from day 3 to day 14.

**Table-1:** Comparison of MAC387 and COX.2 expression.

Time	Group	MAC387	COX2
Day 3	Excision	9.60^a^±3.130	11.60^ac^±0.894
	Excision+ZnO-T	10.00^a^±1.581	10.20^a^±2.588
Day 5	Excision	9.20^a^±1.789	16.20^b^±3.421
	Excision+ZnO-T	17.00^b^±3.082	8.00^c^±1.414
Day 7	Excision	10.60^ac^±1.517	15.20^b^±3.962
	Excision+ZnO-T	16.40^bd^±1.817	6.40^c^±1.673
Day 14	Excision	13.40^abcde^±2.408	11.40^a^±2.966
	Excision+ZnO-T	15.40^bde^±1.673	3.60±1.517

Values are expressed as the mean of five individuals in each group±SD. Means with different superscripts letters are statistically significant at p<0.05. SD=Standard deviation, COX2=Cyclooxygenase2

## Discussion

Macrophages at the wound repair site consist of two populations. The first consists of the resident tissue macrophage permanently present in tissues. Normal skin contains resident macrophages at a low density of approximately 1-2 per mm^2^. The other major population is newly recruited from hematogenous precursor cells known as monocytes. Once newly recruited monocytes migrate through the vessel wall, they release enzymes that fragment extracellular matrix proteins create space for monocytes to migrate into the wound bed. Subsequently, in response to the microenvironment, monocytes differentiate into macrophages. Macrophage numbers increase during the inflammation phase, peaking during the granulation tissue formation phase on day 5 and declining during the maturation phase [11]. In wound granulation tissue, wound-associated macrophages increased until day 2, remained stable until day 5, and then decreased progressively returning to steady-state levels by day14 [12]. MAC387 is able to identify macrophages [6]. Interestingly, the expression trend of macrophages did not mirror that observed in wounds. The expression of MAC387 in the treatment groups increased significantly from day 3 to day 5 and remained high on days 7 and 14. The control group, although stable on days 3 and 5, increased on day 14 because macrophages do not constitute a homogeneous population of cells but exists as multiple phenotypes classified as M1 and M2 phenotypes. The M1 phenotype is pro-inflammatory, phagocytic, and bactericidal, while the M2 macrophages act to inhibit inflammation and regulate revascularization and wound closure. Alternatively, activated M2 macrophages are typically anti-inflammatory. M2 cells were divided *in vitro* into four discrete types - M2a, M2b, M2c, and M2d - based on their function and key markers [13]. Therefore, the high level of macrophage between days 3 and 14 is an anti-inflammatory.

COX-2 was originally discovered as an increased transcription during inflammation and cellular changes [7]. Unwounded epidermis demonstrated the same staining profile as that of normal skin for these COX-1 and -2 proteins. As early as 12 h after injury, COX-2 protein was strongly stained. In addition, on day 10 or after epidermal closure, the distribution and intensity of the staining of these COX proteins returned to a normal pattern in the newly formed epidermis [14]. COX-2 expression in the treatment group of this research showed decreased expression from day 3 to day 14 compared with the control groups that increased on days 5 and 7. This research demonstrated the expression patterns of high COX-2 expression level in the early phase of cutaneous wound healing and the gradually reduced expression, thereafter enabling the tissue to recover from injury. One of the mechanisms of curcumin action as an anti-inflammatory is that of inhibiting the enzyme COX-2, decreasing the production of prostaglandins (causing inflammation), thus accelerating wound healing [8]. Zinc was found to decrease the malondialdehyde level [15], which is also effective as an anti-inflammatory. This research demonstrated that a combination of zinc oxide with turmeric extract has been proven effective as an anti-inflammatory in the healing process of excision wounds.

## Conclusion

The wound dressing combination of zinc oxide and turmeric extract has been proven effective as an anti-inflammatory in the healing process of excision wounds of Wistar rats.

## Authors’ Contributions

We declare that this work was done by the authors named in this article and all liabilities pertaining to claims relating to the content of this article will be borne by them. AM and Aryati designed the experiment; AM, AP, and WR performed the *in vivo* experiment and immunohistochemistry; AM, A, WR, and AP prepared the manuscript. All authors read and approved the final manuscript.
